# Engineered membrane protein antigens successfully induce antibodies against extracellular regions of claudin-5

**DOI:** 10.1038/s41598-018-26560-9

**Published:** 2018-05-30

**Authors:** Yosuke Hashimoto, Wei Zhou, Kohtaroh Hamauchi, Keisuke Shirakura, Takefumi Doi, Kiyohito Yagi, Tatsuya Sawasaki, Yoshiaki Okada, Masuo Kondoh, Hiroyuki Takeda

**Affiliations:** 1 Graduate School of Pharmaceutical Sciences, Osaka University, Osaka, 565-0871 Japan; 2 Proteo-Science Center, Ehime University, Ehime, 790-8577 Japan

## Abstract

The production of antibodies against the extracellular regions (ECR) of multispanning membrane proteins is notoriously difficult because of the low productivity and immunogenicity of membrane proteins due to their complex structure and highly conserved sequences among species. Here, we introduce a new method to generate ECR-binding antibodies utilizing engineered liposomal immunogen prepared using a wheat cell-free protein synthesis system. We used claudin-5 (CLDN-5) as the target antigen, which is a notoriously difficult to produce and poorly immunogenic membrane protein with two highly conserved extracellular loops. We drastically improved the productivity of CLDN-5 in the cell-free system after suppressing and normalizing mRNA GC content. To overcome its low immunogenicity, two engineered antigens were designed and synthesized as proteoliposomes: a human/mouse chimeric CLDN-5, and a CLDN-5-based artificial membrane protein consisting of symmetrically arranged ECRs. Intraperitoneal immunization of both engineered CLDN-5 ECR antigens induced ECR-binding antibodies in mice with a high success rate. We isolated five monoclonal antibodies that specifically recognized CLDN-5 ECR. Antibody clone 2B12 showed high affinity (<10 nM) and inhibited CLDN-5-containing tight junctions. These results demonstrate the effectiveness of the methods for monoclonal antibody development targeting difficult-to-produce membrane proteins such as CLDNs.

## Introduction

Integral membrane proteins are one of the most important classes of proteins in terms of drug targets and biomarkers^1,2^. Binders that recognize the extracellular region (ECR) of membrane proteins are useful for both *in vivo* and *in vitro* applications, such as functional analyses, diagnosis, and therapeutics^3^. Antibodies that recognize and bind the ECR of integral membrane proteins are considered to be canonical binder due to their high specificity and affinity^4^, and ECR-binding antibodies for many targets have been intensively developed by a variety of techniques^5–7^. However, the production of ECR-binding antibodies remains a challenge. This is mainly attributed to the difficulty in the large-scale production of membrane protein antigens and the low immunogenicity due to their complex structures and highly conserved sequences. A representative strategy to raise antibodies against multispanning membrane proteins is proteoliposome immunization^7^. Proteoliposome antigens offer advantages over peptide antigens derived from the extracellular loops of target membrane proteins since peptide antigens are not expected to maintain their correct conformation. The structure of a liposome-embedded membrane protein via its transmembrane domains (TMDs) is much more stable than that of a membrane protein in a micelle particle, which makes proteoliposome immunization an attractive strategy. However, because ECRs are poorly immunogenic due to their compact size and high sequence homology among mammals, a large amount of proteoliposome is required to raise ECR-binding antibodies^6,8^. Proteoliposome production usually requires multiple steps including membrane protein expression, solubilization, purification, and reconstitution, and there are risks of denaturation or loss of target membrane protein in each step^9^.

Previously, we reported a highly efficient production method of proteoliposome using a wheat cell-free protein synthesis system^10^. Cell-free translation is conducted in the presence of high concentration of liposome. The translated multispanning membrane proteins spontaneously associated with liposomes due to their hydrophobicity, and proteoliposome are able to be purified by mild centrifugation^11^. Using this method, membrane proteins can be synthesized with less time and effort. We developed monoclonal antibodies (mAbs) against membrane proteins using cell-free synthesized proteoliposome as an immunizing and screening antigen^10,12^. Although several mAbs against ECRs of multispanning membrane proteins were obtained in these studies, the success rate of extracellular side-recognizing antibody production is low. We have continued the improvement of this antibody development method with cell-free synthesized proteoliposome antigen by generating and characterizing antibodies against the extracellular domain of poorly immunogenic and less-characterized multispanning membrane proteins.

We selected claudin-5 (CLDN-5) as a target membrane protein in this study. CLDN proteins contain four transmembrane domains and two small extracellular loops and comprise 27 family members in human^13^. CLDNs–CLDNs interactions between adjacent cells make tight junctions that regulate paracellular permeability^13^. Because tight junctions are highly detergent-resistant regions in epithelial cells^14^, solubilization techniques for CLDNs from cell-based systems have been poorly developed. Furthermore, methodologies for characterizing CLDN-binders have not been sufficiently developed. In the CLDN family, CLDN-5 attracts special attention since it plays a vital role in the regulation of the blood-brain barrier^15,16^. Although the development of antibodies targeting the ECR of CLDN-5 has been vigorously attempted long time, the production of these antibodies remains to be a challenge. Indeed, there has only been one success report of the production of anti-CLDN-5 ECR mAbs thus far^17^. One reason for the difficulty in antibody production is the remarkably conserved ECR of CLDN-5 among mammals^6^. Knockout mice are often used for raising antibodies against low-immunogenic proteins due to the high sequence homology^18^. However, CLDN-5 knockout mice were not viable and died within 10 h after birth^15^.

In this study, we demonstrate that our improved liposomal immunogen can induce anti-CLDN-5 ECR antibodies with a high success rate. We designed two types of CLDN-5-based artificial membrane proteins for immunization, and synthesized them as proteoliposomes. The liposomes contained lipid-type adjuvant, and purified liposomal immunogens were directly injected into mice without additional procedure. The acquired mAbs showed sufficiently high affinity and selectivity against CLDN-5-expressing cells. Our results reveal a new approach for the generation of mAbs targeting the ECR of poorly immunogenic membrane proteins such as CLDN-5.

## Results

### Preparation of CLDN liposomes using a cell-free translation system

**Figure 1:**
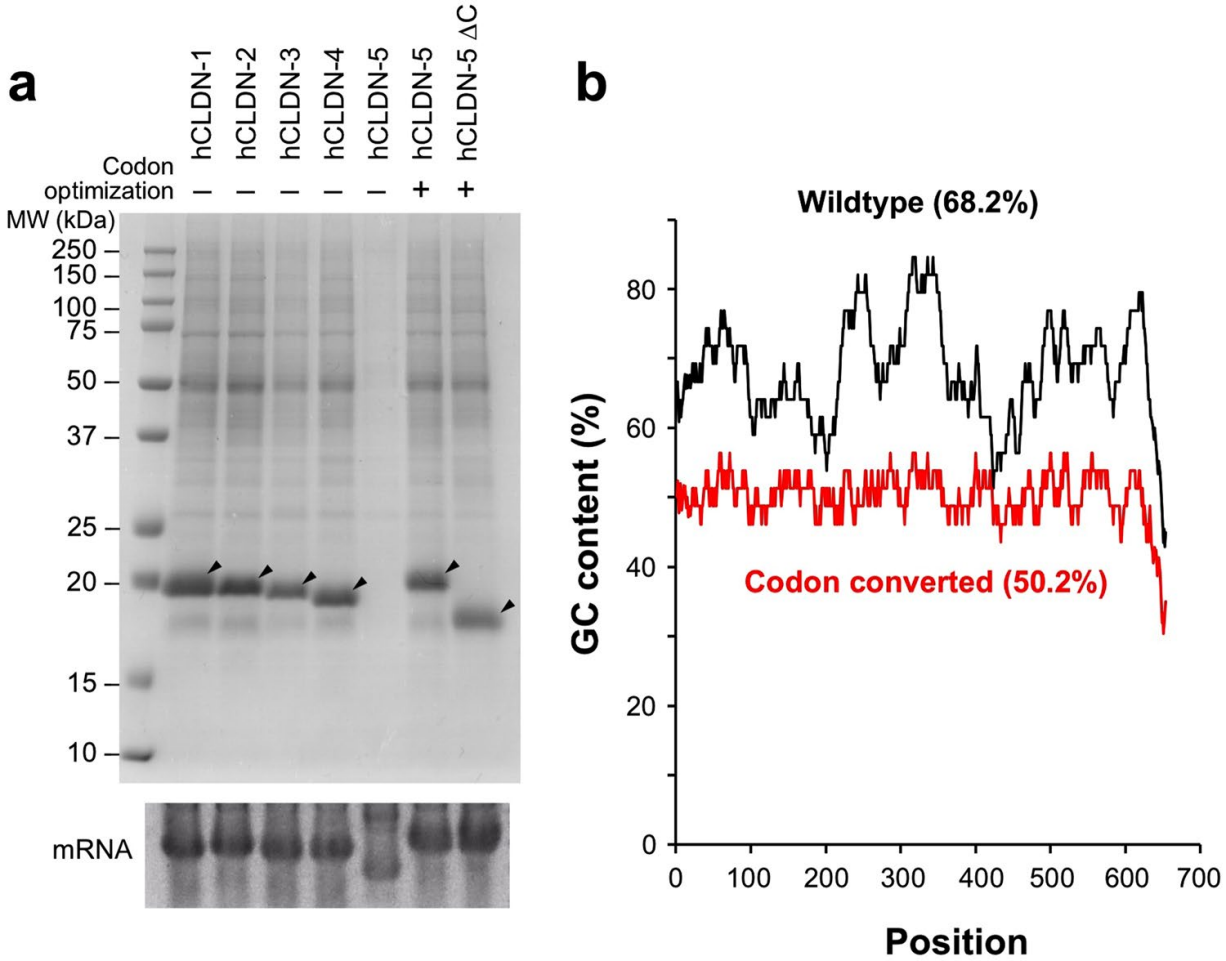
Cell-free synthesis of CLDN-5 with codon-converted mRNA. (**a**) Cell-free synthesized human CLDNs. Human CLDN-1 through CLDN-5 were synthesized on asolectin liposome using the wheat cell-free system with bilayer-dialysis method. After translation, proteoliposomes were purified by repeated centrifugation and resuspension in buffer. Upper panel: purified proteoliposome was subjected to SDS-PAGE and synthesized CLDNs were visualized by CBB staining. Arrowheads indicate translated CLDNs. Lower panel: gel electrophoresis of *CLDN* mRNAs. *In vitro* transcribed mRNA was applied to gel electrophoresis under non-denaturing conditions. (**b**) GC content of wild-type and codon-converted human *CLDN5* mRNAs. The plot indicates local GC content, calculated by averaging 20 bases before and after each position. Figures in parentheses show total GC content. Black, wild-type *CLDN5*; red, codon-converted *CLDN5*.

Previously, we successfully prepared human CLDN-1 through CLDN-4 by the wheat cell-free membrane protein synthesis system in order to evaluate the performance of a binding assay method^10^. Surprisingly, when human CLDN-5 was applied to translation, no CLDN-5 protein was synthesized (Fig. 1a). Since *CLDN-5* mRNA showed faster mobility than the other *CLDN* mRNAs (Fig. 1a, lower panel), we speculated that translation may be hampered by the secondary structure of *CLDN-5* mRNA. The total GC content of *CLDN-5* mRNA was relatively high (68.2%), and repeated GC-rich sequences caused extremely high local GC content (up to 85%) (Fig. 1b). To prevent the folding of *CLDN-5* mRNA, the reduction of the GC content by codon conversion was required^19^. To convert the *CLDN-5* mRNA sequence efficiently, we used GeneArt GeneOptimizer software, a web tool to assist optimization of synthetic genes with codon optimization algorithms for various organisms. *CLDN-5* mRNA was converted using the preferred codon set for *Arabidopsis thaliana* and its total GC content decreased to 50.8% with no obvious high local GC content peaks (Fig. 1b). The codon-converted human *CLDN-5* mRNA showed a similar electrophoresis band pattern to those of other *CLDNs*, and it was successfully translated onto liposomes (Fig. 1a). For immunization, we synthesized 2 mg of C-terminal-truncated CLDN-5 (1–191, Fig. 1a) using an egg phosphatidylcholine (EPC)/monophosphoryl lipid A (MPLA) liposome. The CLDN-5 proteoliposome antigen was immunized subcutaneously at the tail base into 20 BXSB mice, and the induction of ECR-binding antibodies against CLDN-5 was monitored by flow cytometry. However, none of the mice produced antibodies recognizing human CLDN-5 ECR (Table 1).

**Table 1:** Result of immunization experiments.

Antigen	Route	Amount	Induction of anti-CLDN-5 ECR antibodies
Wild-type CLDN-5 ∆C	Tail base	20 µg × 2	0/20
Antigen1	i.p.	20 µg × 4	16/19
Antigen2	i.p.	20 µg × 4	14/17

### Preparation of engineered CLDN-5 antigens for immunization

**Figure 2:**
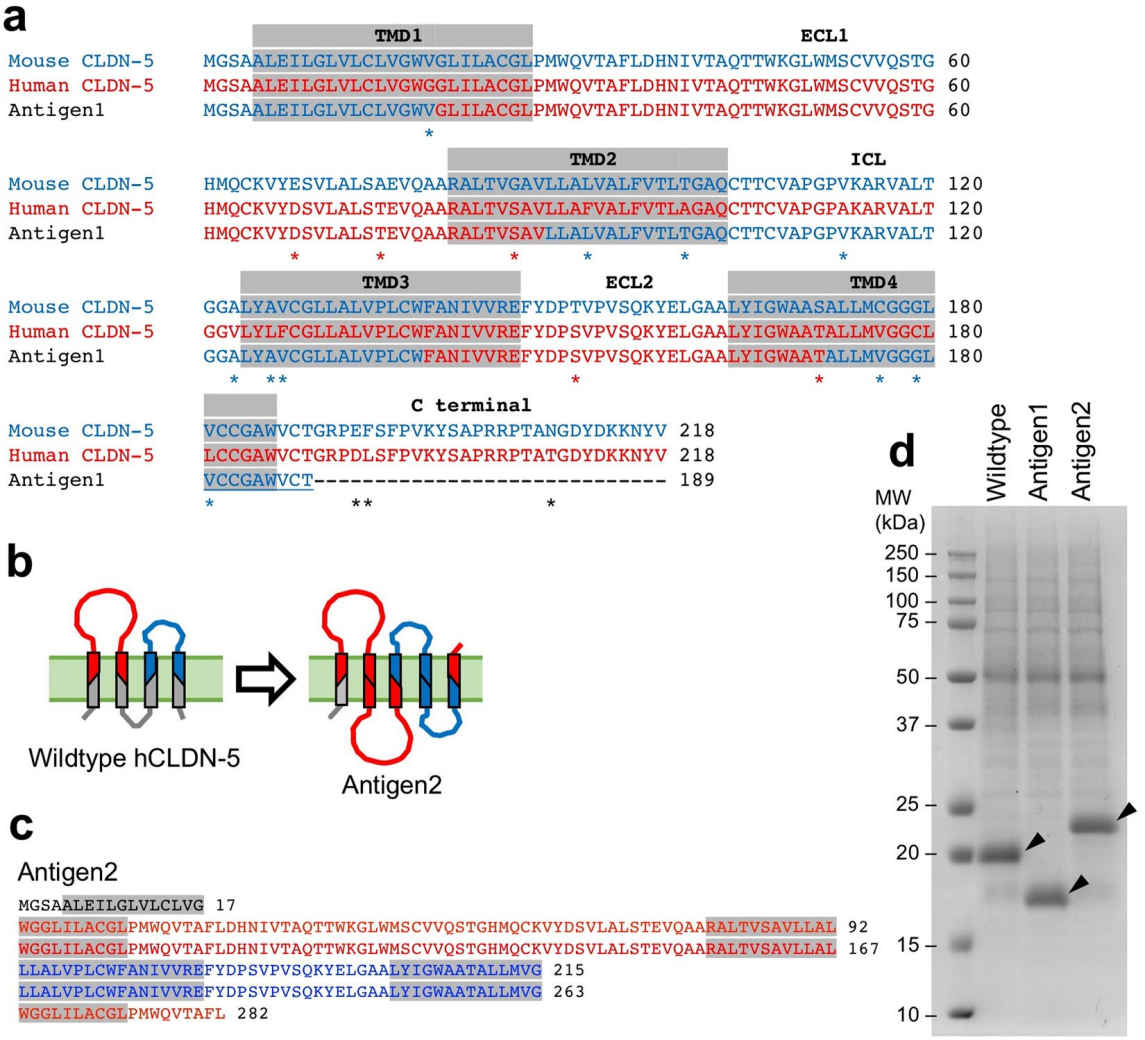
Design of engineered CLDN-5 antigens for immunization. (**a**) Design of Antigen1. Alignment of the amino acid sequences among human CLDN-5, mouse CLDN-5 and human/mouse chimeric CLDN-5 (Antigen1) is shown. Putative TMDs are indicated by gray boxes. Asterisks indicate unmatched amino acids between human and mouse CLDN-5. (**b**) Schematic illustration of Antigen2. Red and blue colors indicate extracellular fragment 1 and 2, respectively. (**c**) Amino acid sequence of Antigen2. Putative TMDs are indicated by gray boxes. Red and blue font colors indicate extracellular fragment 1 and 2 of CLDN-5, respectively. (**d**) Cell-free synthesis of Antigen1 and Antigen2. Purified liposomes were subjected to SDS-PAGE and CBB staining.

To produce ECR-binding antibodies against human CLDN-5 by mouse immunization, we designed two CLDN-5-based membrane protein antigens. First, human/mouse chimeric CLDN-5 (Antigen1, Fig. 2a) was constructed. The ECR of mouse CLDN-5, consisting of two extracellular loops and extracellular-side of TMDs, was substituted with corresponding region of human CLDN-5 amino acid sequence. Twenty-nine amino acids from the C-terminal end of the chimeric CLDN-5 were deleted. Second, we constructed an artificial membrane protein consisting of the ECR of human CLDN-5 (Antigen2, Fig. 2b,c). This antigen contained the N-terminus sequence (7 amino acid residues) with half of TMD1, two tandemly connected first extracellular fragments (the extracellular half of TMD1, the first extracellular loop, and the extracellular half of TMD2), two tandemly connected second extracellular fragments (the extracellular half of TMD3, the second extracellular loop, and the extracellular half of TMD4), the extracellular half of TMD1, and the first 9 residues of the first extracellular loop. Antigen2 was classified as 5-transmembrane protein by TMpred (http://www.ch.embnet.org/software/TMPRED_form.html) and SOSUI programs (http://harrier.nagahama-i-bio.ac.jp/sosui/). Transcription templates of the engineered CLDN-5 ECR antigens were prepared using the gene synthesis service, whereby the GC contents were adjusted using the preferred codon set to *Arabidopsis thaliana* as described above. The antigens were successfully synthesized onto EPC/MPLA liposomes by cell-free translation (Fig. 2d).

Immunization of the engineered CLDN-5 antigens was performed using 20 BXSB mice per antigen. Intraperitoneal immunization of each engineered CLDN-5 ECR antigen successfully induced anti-CLDN-5 ECR antibodies (Table 1). One and three mice died during the immunization period of Antigen1 and Antigen2, respectively.

### Generation of anti-CLDN-5 monoclonal antibodies

**Figure 3:**
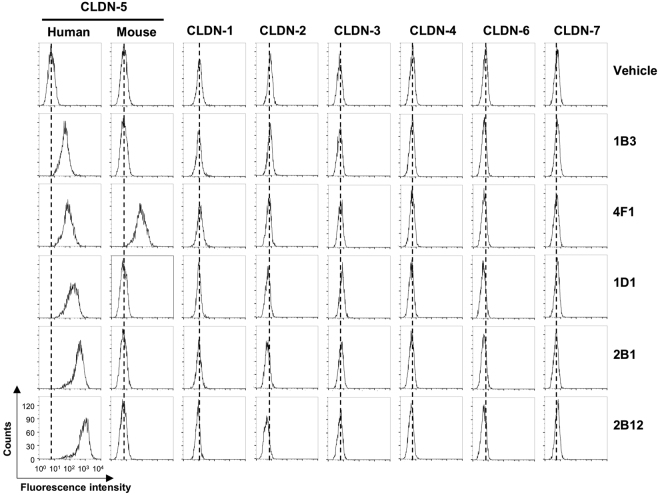
Binding of anti-CLDN-5 ECR mAbs to CLDN–expressing cells. HT-1080 cells expressing CLDNs were treated with vehicle (PBS) or 5 µg/mL of anti-CLDN-5 ECR mAbs. After fluorescein-labeled secondary antibody treatment, cells were analyzed by flow cytometry.

From the immunized mice that produced ECR-binding antibodies against human CLDN-5, five hybridomas producing monoclonal anti-CLDN-5 ECR antibodies were successfully generated. Clone 1B3 and 4F1 were derived from Antigen1-immunized mice, and three additional clones were isolated from Antigen2-immunized mice. We isolated the antibody cDNA clones from the hybridomas and determined the amino acid sequences of their variable domains (Supplementary Fig. S1). The variable domain sequences were confirmed by reconstitution of the recombinant antibodies. We prepared cultured cells expressing human CLDN-1 through CLDN-7, as well as mouse CLDN-5, and investigated the reactivity of anti-CLDN-5 ECR mAbs to these cells using flow cytometry (Fig. 3). All five recombinant antibodies bound to the human CLDN-5-expressing cells. This result indicates that the antibodies were able to recognize and bind to the ECR of CLDN-5. They did not bind to any other human CLDNs. Among the mAbs, only clone 4F1 bound to mouse CLDN-5 orthologue.

**Figure 4:**
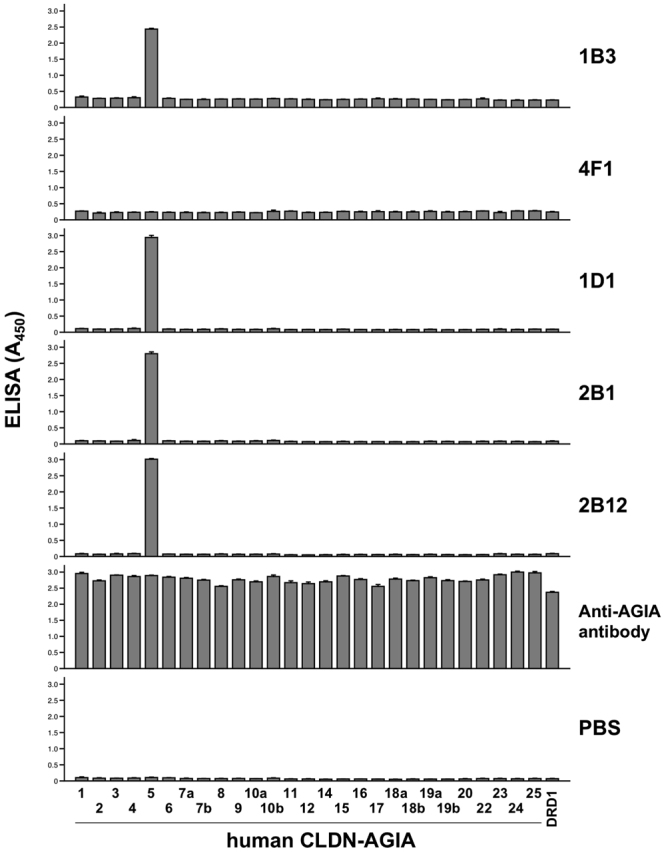
Subtype-specificity analyzed by ELISA with cell-free synthesized CLDN array. Twenty-seven human CLDNs were fused with AGIA tag and synthesized in liposomes by the wheat cell-free system. DRD1 was synthesized as a negative control. Cell-free synthesized proteoliposomes were immobilized on a micro titer plate, and binding between membrane protein and anti-CLDN-5 ECR mAb or anti-AGIA antibody was detected by ELISA.

To fully investigate the subtype specificity of the anti-CLDN-5 ECR mAbs, we established a human CLDN library consisting of all 27 human CLDNs using the cell-free system, and conducted proteoliposome ELISA (Fig. 4). Four out of the five mAbs (all except 4F1) bound only to human CLDN-5, but not to other human CLDNs, demonstrating high subtype specificity. In contrast, clone 4F1 did not bind to any of the cell-free synthesized CLDN proteoliposomes including CLDN-5. There are several differences between cell-expression and cell-free synthesis^20^. For example, glycosylation does not occur in the wheat cell-free system because of the absence of the endoplasmic reticulum and Golgi apparatus in wheat germ extract. Reduced environment in the wheat cell-free translation reaction inhibits the formation of disulfide bonds. These differences may cause artificial conformations of the synthesis product. It is interesting that clone 4F1, which strictly distinguished between cell-expressed CLDN-5 and cell-free synthesized CLDN-5, was induced by the immunization of cell-free synthesized proteoliposome antigen. Some post-immunization events such as oxidization or interaction with other factors may assist in the conformational maturation of the cell-free synthesized CLDN-5.

**Figure 5:**
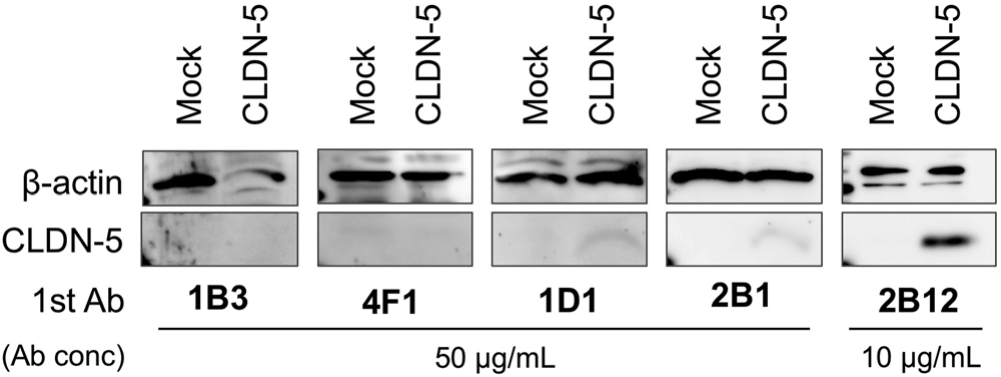
Binding of anti-CLDN-5 antibody to denatured human CLDN-5. Denatured CLDN-5 was detected by western blotting using anti-CLDN-5 ECR mAbs as primary antibody. Lysates of mock or human CLDN-5–expressing cells were subjected to SDS-PAGE. Blotted membranes were incubated with 10 µg/mL 2B12 or 50 µg/mL the other anti-CLDN-5 ECR mAbs. Regions of CLDN-5 band and β-actin band as a loading control were cropped from original blotting images. Full-length blotting images are shown in Supplementary Fig. S2.

We further examined the biochemical features of the monoclonal anti-CLDN-5 antibodies. Clone 2B12 showed the highest affinity for human CLDN-5 (7.37 ± 1.40 nM) among the five mAbs. Clone 2B12 (10 µg/mL) bound to denatured human CLDN-5 in cell lysates separated by sodium dodecyl sulfate-polyacrylamide gel electrophoresis (SDS-PAGE) under reducing condition (Fig. 5). Clone 1B3 and 4F1 (50 µg/mL) did not bind to denatured CLDN-5 at all. Clone 1D1 and 2B1 mAbs barely detected denatured CLDN-5 even at 50 µg/mL. These results suggest that the mAbs, except for 2B12, are sensitive to the conformational changes of CLDN-5. The characteristics of the antibodies generated in this study are summarized in Table 2.

**Table 2:** Summary of monoclonal anti-CLDN-5 ECR mAbs obtained in this study.

Clone	Immunizing antigen	Subtype	Binding specificity			
			hCLDN-5	mCLDN-5	Other hCLDNs	Denatured hCLDN-5
1B3	Antigen1	IgG1/λ	+++	−	−	+
4F1	Antigen1	IgM/κ	+++	+++	−	−
1D1	Antigen2	IgG2a/κ	+++	−	−	+
2B1	Antigen2	IgG2a/κ	+++	−	−	+
2B12	Antigen2	IgG2a/κ	+++	−	−	+++

**Figure 6:**
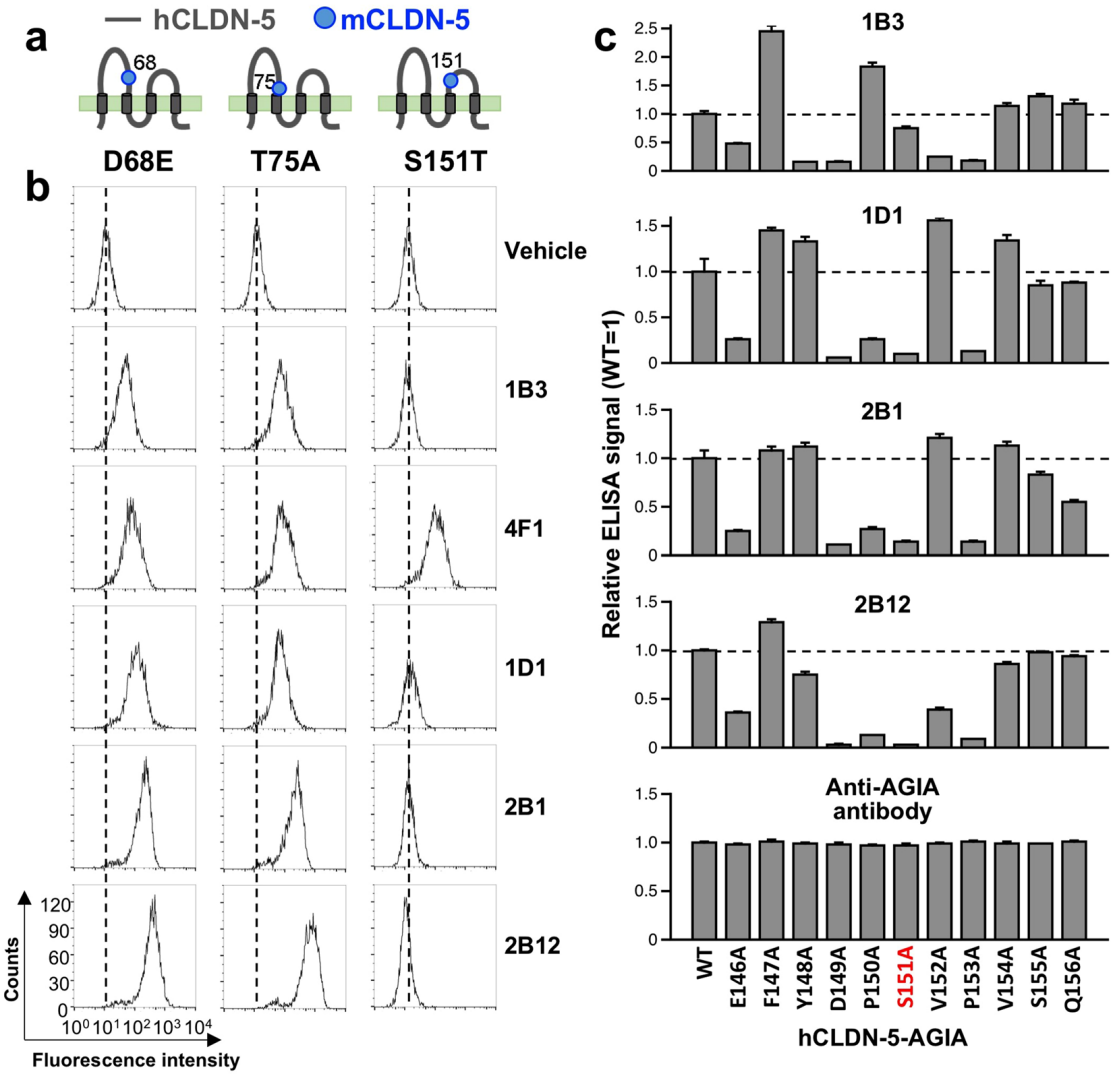
Epitope mapping of anti-CLDN-5 ECR mAbs. Binding reactivity of anti-CLDN-5 ECR mAbs against HT-1080 cells expressing human/mouse CLDN-5 chimera mutants was examined. (**a**) Schematic illustration of the human/mouse CLDN-5 mutants. Single amino acid substitution (D68E, T75A, and S151T) was applied to human CLDN-5. The positions of substitution are indicated by gray dots. (**b**) Flow cytometry. Chimeric mutant expressing cells were treated with vehicle (PBS) or 5 µg/mL of anti-CLDN-5 ECR mAbs. After fluorescein-labeled secondary antibody treatment, fluorescently labeled cells were detected by flow cytometry. (**c**) Alanine scanning. Alanine mutants were constructed using hCLDN-5-AGIA as template. One of the amino acids from E146 to Q156 was substituted by alanine. Mutants were synthesized on liposome by the wheat cell-free system. Cell-free synthesized proteoliposomes were immobilized on a micro titer plate, and binding between membrane protein and anti-CLDN-5 ECR mAb or anti-AGIA antibody was detected by ELISA.

Since most anti-CLDN-5 antibodies did not bind to mouse CLDN-5, we speculated that non-conserved amino acid sequences between human and mouse CLDN-5 may exist in the binding epitope. In the ECR of human CLDN-5, the amino acids D68, T75, and S151 are not conserved in mouse CLDN-5 (Fig. 2a). We prepared three cell lines expressing different CLDN-5 mutants, in which each of the mouse residues were substituted with their respective human residues (D68E, T75A and S151T). We then examined whether mAbs bind to these cells using flow cytometry (Fig. 6a and b). As we expected, clone 4F1, which recognized both human and mouse CLDN-5, bound to all of the chimera mutants. The other 4 antibodies were able to bind to D68E– and T75A–expressing cells but did not bind to S151T–expressing cells. These results indicate that anti-CLDN-5 ECR mAbs, except for 4F1, recognize an epitope containing serine 151 in the second extracellular loop of human CLDN-5. To specifically determine which amino acids in the epitope are recognized by CLDN-5 ECR antibodies, alanine scanning was conducted. One of eleven amino acids around serine 151 was substituted with alanine. Wild-type and mutant CLDN-5 were synthesized using the cell-free method and proteoliposome ELISA was performed (Fig. 6c). The results showed that all antibodies except 4F1 mainly recognized the region from glutamic acid 146 to proline 153. The binding of 1B3 was abrogated by the mutations Y148A, D149A, V152A and P153A. E146A and S151A interfered with the binding of 1B3. Clone 1D1 and 2B1 showed a similar pattern, as they did not bind to the E146A, D149A, P150A, S151A and P153A mutants. 2B1 binding was decreased by half as a result of the Q156A mutation. The binding of 2B12 was abolished by the D149A, P150A, S151A, and P153A mutations, and was also quenched by E146A, Y148A, and V152A.

### The functional activities of anti-CLDN-5 antibodies

**Figure 7:**
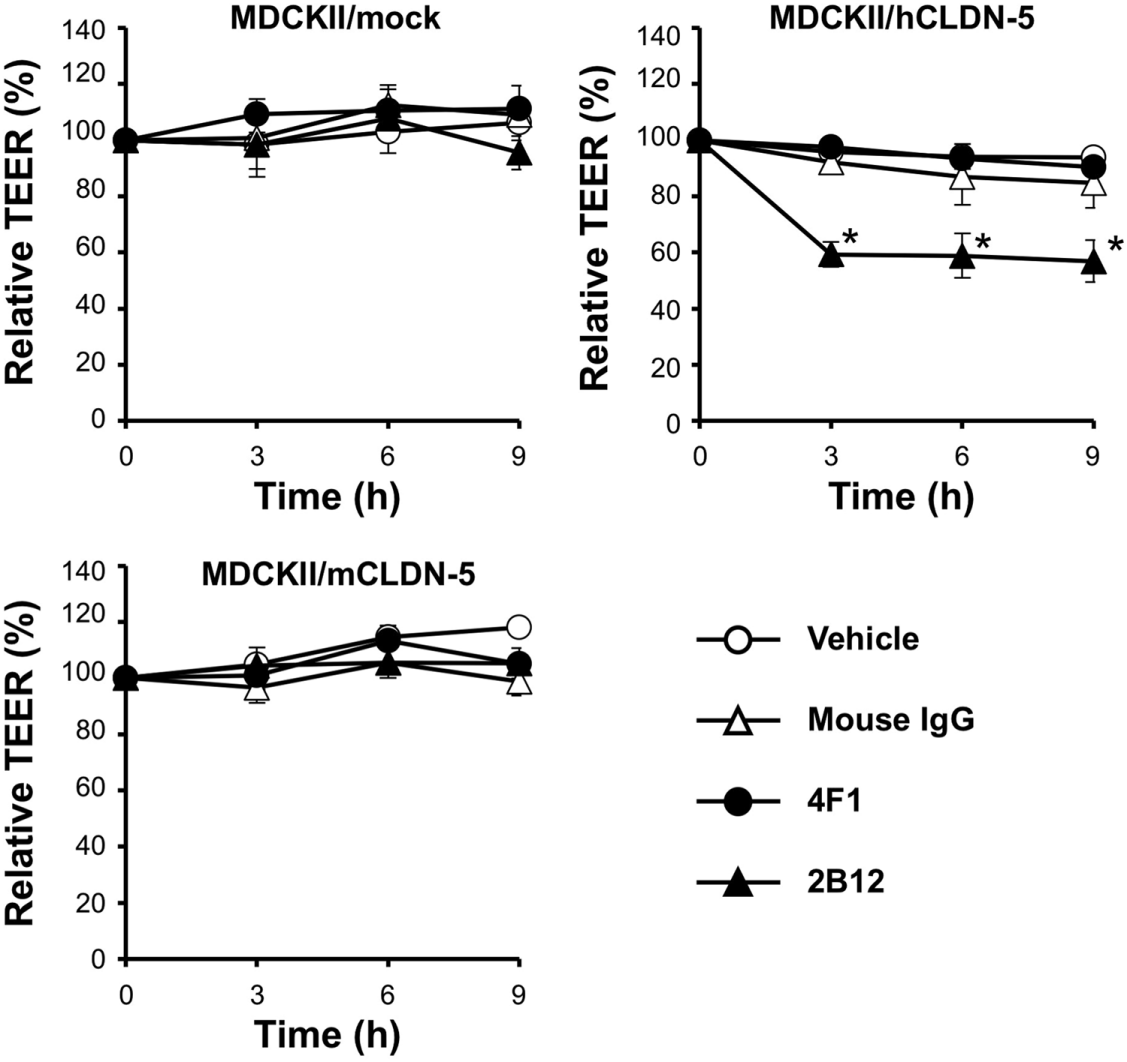
Effect of anti-CLDN-5 ECR mAbs on the paracellular barrier of CLDN-5–expressing cells monolayer. Monolayers of MDCKII expressing mock (upper left), human CLDN-5 (upper right) or mouse CLDN-5 (lower left) were basolaterally treated with vehicle (PBS) or 135 µg/mL of antibodies (mouse IgG, 4F1 or 2B12) for 9 h. TEER was monitored throughout the treatment period. Relative TEER (%) to the value at 0 h is shown. Error bars represent standard error of the mean values (n = 3). *P < 0.05 versus vehicle treatment.

To investigate whether the generated anti-CLDN-5 ECR antibodies inhibit the function of CLDN-5, we focused on the homophilic interactions of CLDN-5 that form the intercellular barrier^13,21^. We used a trans-epithelial/endothelial electrical resistance (TEER) assay of CLDN-5–based intercellular barrier, which indicates the strength of intercellular barrier, using human or mouse CLDN-5-expressing MDCKII cells. Treatment with the anti-CLDN-5 ECR mAb clone 2B12 significantly decreased TEER in the hCLDN-5–expressing MDCKII cell monolayer (Fig. 7). The mouse CLDN-5 barrier was not affected by 2B12. In contrast, clone 4F1 did not modulate either the human or mouse CLDN-5–based barrier, although 4F1 did bind to both human and mouse CLDN-5–expressing cells (Fig. 3).

We further characterized the inhibitory activity of clone 2B12. We confirmed that 2B12 binds to cynomolgus monkey CLDN-5 using flow cytometry (Supplementary Fig. 3a), and performed TEER assay using a commercially available monolayer of brain microcapillary endothelial cells from cynomolgus monkeys^22^. Treatment with 2B12 decreased the paracellular barrier of the cell monolayer in a time-dependent manner (Supplementary Fig. 3b). These results imply that the anti-CLDN-5 ECR mAb 2B12 binds to an epitope around the region from E146 to P153 and has is able to modulate the barrier formation activity of CLDN-5.

## Discussion

Wheat cell-free translation system can synthesize multispanning membrane proteins onto liposomes with high efficiency and throughput, and is suitable for preparing large amounts of antigen for immunization^10,23^. In our current study, we have applied several modifications in antigen production and immunization methods to enhance the induction of ECR-binding antibodies. These alterations successfully induced anti-CLDN-5 ECR antibodies in mice following immunization of cell-free synthesized proteoliposome antigen.

Firstly, we suppressed and normalized the GC content in *CLDN-5* mRNA and remarkably improved CLDN-5 expression in the wheat system (Fig. 1). Originally, the activity and efficacy of the translation machinery in wheat germ extract is rarely affected by codon usage bias^24^. Indeed, this system has efficiently produced thousands of human, animal, plant, viral, and malarial proteins without codon optimization^25^. It has also expressed dozens of multispanning membrane proteins, including G protein-coupled receptors, transporters and CLDNs^10,11^. Surprisingly, however, it failed to synthesize human CLDN-5 (Fig. 1a). The total GC content of wild-type human *CLDN-5* mRNA is as high as 68%, and local GC content was further increased to 85%, because of repeated GC-rich sequences (Fig. 1b). We speculate that *CLDN-5* mRNA with high GC content forms complex conformations *in vitro*, which inhibits the movement of ribosomes on mRNA by steric hindrance. Indeed, CLDN-5 was successfully synthesized (Fig. 1a) after we modified the template DNA sequence using GeneOptimizer application, in which the total GC content was reduced to 50.2% and the local GC content was limited to the range of 45% to 55% (Fig. 1b). We used the codon set for *Arabidopsis thaliana* to decrease GC content because its algorithm suppressed and normalized GC content of *CLDN-5* mRNA better than others. GeneOptimizer also contains an algorithm for Barley (*Hordeum vulgare*), which is also a monocot plant and phylogenetically closer to wheat than *Arabidopsis*. However, it did not suppress the average GC content of human *CLDN-5* to the extent of *Arabidopsis*, and was not selected for this reason.

Secondly, we modified the liposome composition for immunization. In our previous reports, we synthesized membrane proteins using asolectin liposome, and prepared emulsion by mixing Freund’s adjuvant and proteoliposomes prior to immunization^10^. In the current report, MPLA, a lipid adjuvant, was mixed with EPC in advance of liposome preparation, and CLDN-5 antigens were synthesized in the adjuvant-containing liposome. We injected the adjuvant-containing proteoliposomes into mice, and successfully induced immune responses and antibody production in the mice. Vigorous and prolonged mixing to form an antigen-adjuvant emulsion would be too harsh for delicate membrane proteins. Adjuvant-containing liposomes may provide milder condition for membrane proteins, contributing to the retention of their conformation.

Thirdly, we designed novel artificial membrane proteins, which maximized the possibility to induce CLDN-5 ECR-binding antibodies. Human CLDN-5 has 92% similarity to the mouse ortholog, and the antigenicity of human CLDN-5 is quite low. We engineered two CLDN-5-based membrane proteins for immunization. Until now, it is not possible to control the topology of cell-free synthesized membrane proteins on a liposome^10^, and both extracellular and intracellular regions are equally exposed to the immune system. Induction of antibodies against the intracellular region of CLDN-5 in immunized mice would interfere the ECR-binding antibody production. Therefore, it is advantageous if the intracellular regions of CLDN-5 antigen are concealed or eliminated in advance. Antigen1 was a human/mouse chimera of CLDN-5, with extracellular and intracellular regions composed of human and mouse CLDN-5 sequences, respectively (Fig. 2a). When mice were immunized with Antigen1, the production of antibodies recognizing the intracellular region of CLDN-5 was expected to be suppressed by immune tolerance. Antigen2 was designed with a more ambitious approach, in which the ECR was symmetrically arranged on both sides (Fig. 2c). Although high structural integrity of the CLDN-5 ECR cannot be expected, Antigen2 without the CLDN-5 intracellular region theoretically induces the production of ECR-binding antibodies only. Both antigens induced immune responses with a high rate of success (Table 1), and produced functional antibodies. It was reported that it is sometimes difficult to correctly express engineered membrane proteins on the cell surface under normal cellular processes in mammalian cells^26^. However, both of these membrane proteins were successfully synthesized to the same extent as native CLDN-5 by the cell-free system (Fig. 2d). Thus, cell-free synthesized artificial membrane proteins may provide new choices for antigen preparation.

To overcome the challenges of inducing mAb production against low-immunogenic CLDN-5 ECR, further innovations in antigen design are required. Our cell-free synthesized proteoliposomes provide several advantages in immunization. First, our liposomal immunogens contained a large quantity of antigens. Highly dense antigens have been shown to strongly activate B cells via crosslinking with B cell receptors^27^. Our liposomes also contained MPLA, a toll-like receptor 4 agonist. Co-engagement of toll-like receptor 4 and B cell receptor by a formulated immunogen enhances antibody production^27,28^. Thus, intraperitoneal injection of our MPLA-containing liposomal immunogens would allow immunogens to directly interact with peritoneal or splenic B cells. In addition, the stimulation of toll-like receptor 4 agonist accelerates lupus symptoms of BXSB mice, resulting in a decreased activation threshold of B cells^29^. We believe that the combination of these innovations resulted in the enhanced production of anti-CLDN-5 antibodies.

We have developed several new approaches to produce anti-CLDN-5 ECR antibodies, including cell-free synthesized proteoliposome antigen, the improvement of CLDN-5 expression by codon-optimization, the use of MPLA-containing liposomes, and the design of new antigens. The ECR mAbs obtained in this study were specific to human CLDN-5 and did not cross-react with mouse orthologs, except for clone 4F1. Most mAbs were sensitive to the conformation of CLDN-5 (Fig. 3d). It should also be noted that clone 2B12, which had highest affinity to CLDN-5, showed inhibitory activity on the CLDN-5–based barrier (Fig. 5, Supplementary Fig. 3). Our results demonstrate that these approaches are able to generate functional mAbs recognizing human CLDN-5 ECR. There are a multitude of difficult-to-express membrane protein targets with small and low immunogenic ECR, similar to CLDN-5. Currently it is difficult to develop antibodies against these types of antigens. We hope that the approaches examined in this study will be applied to solve some of the problems involved in the generation of antibodies against difficult targets.

## Methods

### Animals

Male BXSB mice (6 weeks old) and female BALB/c nu/nu mice were purchased from Shimizu Laboratory Supplies (Kyoto, Japan). All mice were maintained under controlled conditions with a 12:12 h light: dark cycle at 23 ± 1.5 °C. Mice were given *ad libitum* access to food and water. Animal experiments were approved by Osaka University (Osaka, Japan), and performed in accordance with the guidelines.

### DNA

cDNAs encoding human genes of CLDN-1 to -25 were amplified from the Mammalian Gene Collection full-length cDNA clone set^30^ or synthesized using gene synthesis (GeneArt, Regensburg, Germany) (Table S1). Codon optimization of CLDN-5 sequence was performed by using GeneOptimizer (GeneArt) to lower and normalize the extremely high GC content of the synthesized genes^19^. DNA fragments of CLDN-5 ECL antigen Antigen1 and Antigen2 were also synthesized. For non-tagged protein synthesis, cDNA fragments were inserted into the pEU-E01-GW vector using Gibson Assembly (New England Biolabs, Beverly, MA). For AGIA tag fusion protein expression, pEU-E01-AGIA-GW vector containing a start codon and an AGIA-tag at the 5′-end of the gateway cassette was used^31^. Alanine mutants were prepared using PrimeSTAR Mutagenesis Basal Kit (Takara Bio, Shiga, Japan). Plasmids were purified by NucleoBond Xtra midi kit (Macherey-Nagel, Duren, Germany).

### Preparation of liposomes

Asolectin was purchased from Sigma-Aldrich (St. Louis, MO) and purified by acetone and chloroform as previously reported^23^. EPC was purchased from Wako Pure Chemicals (Osaka, Japan) and synthesized MPLA was purchased from Avanti Polar Lipids (Alabaster, AL). These lipids were dissolved in chloroform at 100 mg/mL (asolectin and EPC) or 10 mg/mL (MPLA). For preparation of the asolectin liposome, 100 mg of asolectin was evaporated to form a thin lipid film using a vacuum rotary evaporator (N-1110, EYELA, Tokyo, Japan). For the EPC/MPLA liposome, 100 mg of EPC and 2.5 mg of MPLA were mixed and evaporated as well. Fully dried thin lipid films were hydrated by 1 mL (asolectin liposome, 100 mg lipids/mL) or 4 mL (EPC/MPLA liposome, 25 mg/mL) of SUB-AMIX SGC solution (CellFree Sciences, Ehime, Japan) with mild sonication using a SONIFIER model 450 Advanced (BRANSON, Danbury, CT).

### Wheat Cell-free synthesis of CLDN proteoliposomes

Cell-free synthesis of CLDN proteoliposomes was performed by bilayer-dialysis method as described previously with minor modifications^10^. *In vitro* transcription was performed using SP6 RNA polymerase (Promega, Madison, WI). The transcribed RNA was analyzed by agarose gel electrophoresis using non-denatured Tris acetate- ethylenediaminetetraacetic acid (EDTA) buffered gel. The RNA was visualized by SYBR safe dye (Thermo Fisher Scientific). Five hundred µL of translation reaction mixture containing mRNA (25%), WEPRO 7240 wheat germ extract (25%) (CellFree Sciences), creatine kinase (40 μg/mL) (Roche diagnostics, Mannheim, Germany) and liposomes (10 mg lipids/mL) was overlaid with 2 mL of SUB-AMIX SGC solution in a 10-K MWCO Slide-A-Lyzer MINI dialysis device (Thermo Fisher Scientific), the cup was then immersed in 3.5 mL dialysis solution (SUB-AMIX SGC). Asolectin liposomes were used for examining protein expressions and EPC/MPLA liposomes were used for preparation of immunogens. The reaction was carried out at 16 °C for 24 h.

A cell-free synthesized proteoliposome was collected by centrifugation at 20,000 × g for 10 min at 4 °C, and a resultant pellet was suspended in sterile phosphate-buffered saline (PBS). Centrifugation and suspension cycles were repeated 3 times. The pellet was finally re-suspended in a small amount of sterile PBS and the concentration of synthesized CLDNs on liposomes was estimated by SDS-PAGE with Coomassie brilliant blue (CBB) staining with bovine serum albumin as a standard. Proteoliposome suspension was fractioned into small portions, frozen by liquid nitrogen and stored at −80 °C.

### Cells

HT-1080 cells stably expressing mock (empty vector), human CLDN-1 to -7 and mouse CLDN-5 were developed as described previously^32^. MDCKII and P3U1 cells were purchased from ATCC (Manassas, VA). HT-1080 cells expressing human CLDN-5 mutants (D68E, T75A and S151T) and MDCKII cells expressing human or mouse CLDN-5 were prepared in a similar way as described previously^32^.

HT-1080 and MDCKII cells were maintained in Dulbecco’s modified Eagle’s medium (DMEM) supplemented with 10% heat-inactivated fetal bovine serum (FBS) (v/v) (Nichirei Biosciences, Tokyo, Japan), 100 U/mL penicillin, and 100 µg/mL streptomycin (Nacalai Tesque, Kyoto, Japan). P3U1 cells were maintained in RPMI1640 medium supplemented with 10% heat-inactivated FBS, 100 U/mL penicillin, and 100 µg/mL streptomycin. All hybridomas were maintained in 20% heat-inactivated FBS, 10% BM Condimed H1 (Roche Diagnostics), 100 U/mL penicillin, and 100 µg/mL streptomycin. All cells were incubated under an atmosphere of 5% CO_2_ in air at 37 °C.

### Generation and purification of anti-CLDN-5 ECR mAbs

Male BXSB mice were immunized intraperitoneally or subcutaneously at tail base every 2 weeks for 8 weeks with the EPC/MPLA liposomes containing 20 µg of Antigen1 or Antigen2. To generate hybridoma cells, splenocytes were harvested 60 h after the final immunization and fused with P3U1 cells using polyethylene glycol 1500 (Roche Diagnostics). Hybridoma cells producing anti-hCLDN-5 ECR antibodies were screened by flow cytometry using HT-1080/human CLDN-5 and HT-1080/mock cells as described below. The mAb subclass was determined using the IsoStrip Mouse Monoclonal Antibody Isotyping Kit (Sigma-Aldrich).

Purified mAbs were prepared as described previously^32^. Briefly, hybridoma cells were inoculated intraperitoneally into pristane-injected female BALB/c nu/nu mice, resulting in the production of ascitic fluid containing the desired mAbs. The IgG fraction was purified from the ascitic fluid using Protein G Sepharose 4 Fast Flow (GE Healthcare, Princeton, NJ). The purified mAb was then dialyzed against PBS and stored at −30 °C.

### Flow cytometric analysis

For binding analysis, cultured cells expressing various CLDNs were detached from culture plates by using 0.05% trypsin containing 0.02% EDTA. The cells were then incubated with a serum, a culture supernatant, or a purified mAb (5 μg/mL), and then stained with fluorescence-conjugated goat anti-mouse IgG (Jackson ImmunoResearch, West Grove, PA). To determine the affinity of the generated mAbs to hCLDN-5, duplicate aliquots of HT-1080/hCLDN-5 cells were treated with a purified anti-CLDN-5 ECR mAb (0.0024–40 μg/mL). Fluorescence intensity of the stained cells was determined using FACSCalibur flow cytometer (BD Biosciences, San Diego, CA). Mean fluorescence intensity values were used to obtain the apparent dissociation constant of an anti-CLDN-5 ECR mAb, which was determined by means of nonlinear regression with an on-site binding model according to the following equation: Y = B_max_ • X/ (K_d_ + X). Calculations were performed with GraphPad Prism version 7.0 (GraphPad Software, San Diego, CA). Data are presented as the mean ± SD of three independent experiments.

### Western blotting

To examine binding of acquired anti-claudin-5 mAbs to denatured human CLDN-5, lysates of HT-1080/mock and HT-1080/human CLDN-5 cells were separated by SDS-PAGE under reducing condition and then transferred to polyvinylidene difluoride membranes. After blocking with 5% skimmed milk containing Tris-buffered saline with 0.05% tween-20 (T-TBS), the membranes were incubated with 10 µg/mL of 2B12 or 50 µg/mL of the other anti-CLDN-5 ECL mAbs in T-TBS containing 2% skimmed milk for 12 h at 4 °C. Mouse monoclonal anti-β-actin (AC-15, Sigma-Aldrich) was used as the loading control. After incubation of the primary antibodies, the membranes were incubated with a horseradish peroxidase–conjugated anti-mouse IgG + IgM secondary antibody (Jackson ImmunoResearch) in T-TBS and then incubated for 2 h at room temperature. After extensive washes with T-TBS, the membranes were treated with Chemi-Lumi Super (Nacalai Tesque) to detect the horseradish peroxidase, and chemiluminescent signals were acquired using LAS4100 imager (GE Healthcare, Princeton, NJ).

### ELISA

Cell-free synthesized proteoliposomes of AGIA tag fusion CLDNs were diluted 20-folds and injected to a 96 well MaxiSoap microliter well plate (Nunc, Roskilde, Denmark). Proteoliposome of dopamine receptor D1 (DRD1), which contains AGIA epitope sequence^31^, was used as a negative control. Immobilization of CLDNs was performed by incubating overnight at 4 °C. After washing by T-TBS, the plate was blocked with 5% skimmed milk in T-TBS for 1 h at room temperature. The plate was incubated with an anti-CLDN-5 ECR mAb in 5% skimmed milk/T-TBS at 10 µg/mL concentration for 1 h at room temperature. The anti-AGIA tag antibody was used for monitoring the amount of CLDNs. After incubation of the primary antibodies, the plate was washed by T-TBS for three times, then incubated with a horseradish peroxidase–conjugated anti-mouse IgG + IgM secondary antibody (Jackson ImmunoResearch) in 5% skimmed milk/T-TBS and then incubated for 1 h at room temperature. Finally, 50 µL of tetramethylbenzidine liquid substrate (Sigma-Aldrich) was injected to the plate; incubate for 15–30 min at room temperature. The reaction was terminated by injecting same volume of 1 M HCl, and absorbance at 450 nm was measured by SpectraMAX M3 plate reader (Molecular Devices, Sunnyvale, CA).

### Measurement of the electrical resistance of cell monolayers

TEER of cell monolayer, which reflects the integrity of the barrier in tissues, was measured using Millicell ERS Ohmmeter (Millipore, Eschborn, Germany) on a culture plate warmer. MDCKII/mock, MDCKII/hCLDN-5 or MDCKII/mCLDN-5 cells were cultured on a culture inserts with 0.4 µm pore until the TEER value reached plateau. After the exchange of 90 μL of medium from the lower compartment and 90 µL of antibody solution (1350 µg/mL, final concentration at 135 µg/mL), TEER was measured for 9 h.

### Statistical analysis

Data were analyzed by Student’s *t*-test followed by a post hoc pairwise comparison. Statistical significance for all comparisons was set at *P* < 0.05.

### Data availability statement

All data generated or analysed during this study are included in this published article and its Supplementary Information files.

## Electronic supplementary material

Supplementary Information
