# Supplementary material for: Engineered membrane protein antigens successfully induce antibodies against extracellular regions of claudin-5
Source: Sci Rep. 2018 May 30;8:8383. doi: 10.1038/s41598-018-26560-9 (PMC5976803; doi:10.1038/s41598-018-26560-9)
Supplement: Supplementary file 1 — Supplementary Information [file 41598_2018_26560_MOESM1_ESM.pdf]

Supplementary Information

Engineered membrane protein antigens successfully induce antibodies against extracellular regions of claudin-5.

Yosuke Hashimoto[1], Wei Zhou[2], Kotaro Hamauchi[2], Keisuke Shirakura[1], Takefumi Doi[1], Kiyohito Yagi[1], Tatsuya Sawasaki[2], Yoshiaki Okada[1], Masuo Kondoh[1*], Hiroyuki Takeda[2*].

Supplementary Methods

Determination of amino acids sequence of anti-claudin-5 antibodies. mRNAs of anti-CLDN-5 ECR mAbs were isolated from hybridomas using Sepasol-RNA I Super G (Nacalai Tesque, Kyoto, Japan), and first strand cDNA was synthesized by Transcriptor First Strand cDNA Synthesis Kit (Roche Diagnostics, Mannheim, Germany). cDNAs of variable heavy-chain (VH) and variable light-chain (VL) domains were amplified by PCR using mouse-Ig primer kit (Novagen, Darmstadt, Germany) and then inserted into the pUC18 vector. Sequencing was conducted by Fasmac (Yokohama, Japan).

To confirm the amino acids sequence of the antibodies, DNA fragments of VH and VL domains were connected with corresponding constant domains prepared by gene synthesis, and were subcloned into the pcDNA3.4 vector (Thermo Fisher Scientific, Waltham, MA) using Gibson Assembly. Both pcDNA3.4-IgG heavy chain and pcDNA3.4-IgG light chain were co-transfected into Expi293F (Thermo Fisher Scientific) and cultured for 7 days. The binding reactivity of the recombinant anti-CLDN-5 mAbs in culture supernatants against hCLDN-5 expressing cells was analyzed by flow cytometric analysis.

Flow cytometric analysis. To analyze binding specificity of anti-CLDN-5 ECR mAb clone 2B12 to cynomolgus monkey CLDN-5, HT-1080 cells stably expressing cynomolgus monkey CLDN-5 (accession number XP_005596069) were prepared. Cells expressing human CLDN-5 or cynomolgus monkey CLDN-5 were detached from the culture plates by using 0.05% trypsin containing 0.02% ethylenediaminetetraacetic acid. The cells were then incubated with 2B12 (5 µg/mL) and stained with fluorescence-conjugated goat anti-mouse IgG (Jackson ImmunoResearch, West Grove, PA). Fluorescence intensity of the stained cells was determined using a FACSCalibur flow cytometer (BD Biosciences, San Diego, CA).

Measurement of the electrical resistance of cell monolayers. To investigate the effect of anti-CLDN-5 ECR mAb clone 2B12 to monolayer of cynomolgus monkey brain microvasculature cells, trans-epithelial/endothelial electrical resistance (TEER) was measured using Millicell ERS Ohmmeter (Millipore, Eschborn, Germany) with a culture plate warmer. In the advance of TEER assay, monkey BBB triple co-culture model kit (MBT-24F, PharmaCo-cell, Nagasaki, Japan) was cultured for 5 days. After the exchange of 30 µL of medium from the upper compartment with 30 *µ*L of PBS (vehicle), 5000 *µ*g/mL of mouse IgG, or 2B12 (final concentration, 500 *µ*g/mL), the TEER of monolayer was measured for 9 h.

Statistical analysis. Data were analyzed by using Student's *t*-test followed by a post hoc pairwise comparison. Statistical significance for all comparisons was set at $P < 0.05$.

a

	Frame 1	**CDR 1**	Frame 2	**CDR 2**
1B3	QIQLVQSGPELKKPGETVKISCKAS	**GYSFTAH**	GMSWVKQAPGKGLKWMGWI	**NTYSGV**
4F1	QVQLQQSGPELVKPGASVKISCKAS	**GYSFTSY**	YIHWVKQRPGQGLEWIGWI	**YPGSGN**
1D1	QVQLQQPGAEHVKPGASVKLSCKAS	**GYTFTTY**	WIHWVKQRPGRGLEWIGRI	**APNSGG**
2B1	QVQLQQPGAELVKPGASVKLSCKAS	**GYTFTTF**	WIHWVKQRPGRGLEWIGRI	**APYSGG**
2B12	QVQLQQSGAELARPGASVKLSCKAS	**GYTFTRF**	GMSWVKQRTGQGLEWIGEI	**YPGSGD**

	Frame 3	**CDR 3**	Frame 4
1B3	PAYADDFKGRFAFSLETSPSTAFLQINNLKNEDTATYFCTR	**SHYDR----KFGY**	WGQGTLVTVSA
4F1	TKYNEKFKGKATLTADTSSSTAYMQLSSLTSEDSAVYYCAS	**PYYGS-RRDYFDY**	WGQGTTLTVSS
1D1	TKYNENFKSKATLTVDRPSTTAYMQLSGLTSEDSAVYYCAR	**WDFT--FGTNLDY**	WGQGTTLTVSS
2B1	TTYNEKFKSKATLTVDRPSTTAYMQLISLTSEDSAVYYCAR	**WDFT--YGSNLDY**	WGQGTTLTVSS
2B12	TYYSENFKGKATLTADKSSGTAYMELRSLTSEDSAVYFCAR	**WGIYYGNPYAMDY**	WGQGTSVTVSS

b

	Frame 1	**CDR 1**	Frame 2	**CDR 2**
1B3	QAVVTQES-ALTTSPGETVTLTC	**RSSTG---AVTTSNYAN**	WVQEKPDHLFTGLIG	**DTNNRAP**
4F1	DIVMSQSPSSLAVSVGEKVTMSC	**KSSQSLLYSSNQKNYLA**	WYQQKPGQSPKLLIY	**WASTRES**
1D1	DIVMTQSQKFMSTSVGDRVSITC	**KAS------QNVRTAVA**	WYQQKPGQSPKALIF	**LASNRHT**
2B1	DIVMTQSQKFMSTSVGDRVSIPC	**KAS------QNVRTAVA**	WYQQKPGQSPKALIY	**LASNRHT**
2B12	DIVMTQAAPSVPVTPGESVSISC	**RSSKS-LLHSNGNTYLY**	WFLQRPGQSPQLLIY	**RMSNLAS**

	Frame 3	**CDR 3**	Frame 4
1B3	GVPARFSGSLIGDKAALTITGAQTGDEAIYFC	**ALWYSNLWV**	FGGGTKLTVRGQP
4F1	GVPDRFTGSGSGTDFTLTISSVKAEDLAVYYC	**QQYYSYPLT**	FGAGTKLELKRA-
1D1	GVPDRFTGSGSGTDFTLTITNVQSEDLADYFC	**LQHWTYPYT**	FGGGTKLEIKRA-
2B1	GVPDRFTGSGSGTDFTLTISNVQSEDLADYFC	**LGHWDYPYT**	FGGGTKLEIKRA-
2B12	GVPDRFSGSGSGTAFTLRISRVEAEDVGVYYC	**LQHLEYPFT**	FGSGTKLEIKRA-

Supplementary Figure S1. Amino acid sequences of variable domain of anti-CLDN-5 ECR mAbs. Amino acid sequences of VH (**a**) and VL (**b**) domains are shown, respectively. Complementarity determining regions (CDRs) are represented in bold typeface.

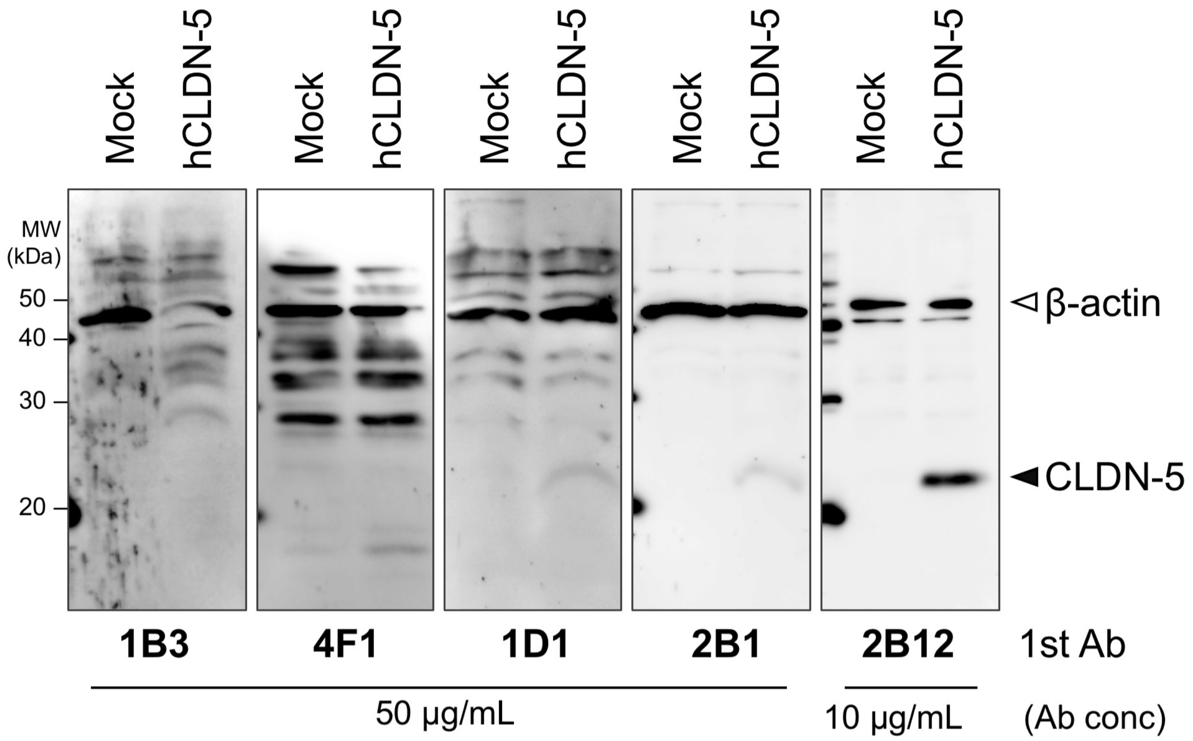

Supplementary Figure S2. Western blotting. Full-length blotting image of Figure 5. Lysates of mock or human CLDN-5–expressing cells were subjected to SDS-PAGE. Blotted membranes were incubated with 10 *μ*g/mL 2B12 or 50 *μ*g/mL the other anti-CLDN-5 ECR mAbs. β-actin was detected in the same blot, respectively, and used as a loading control.

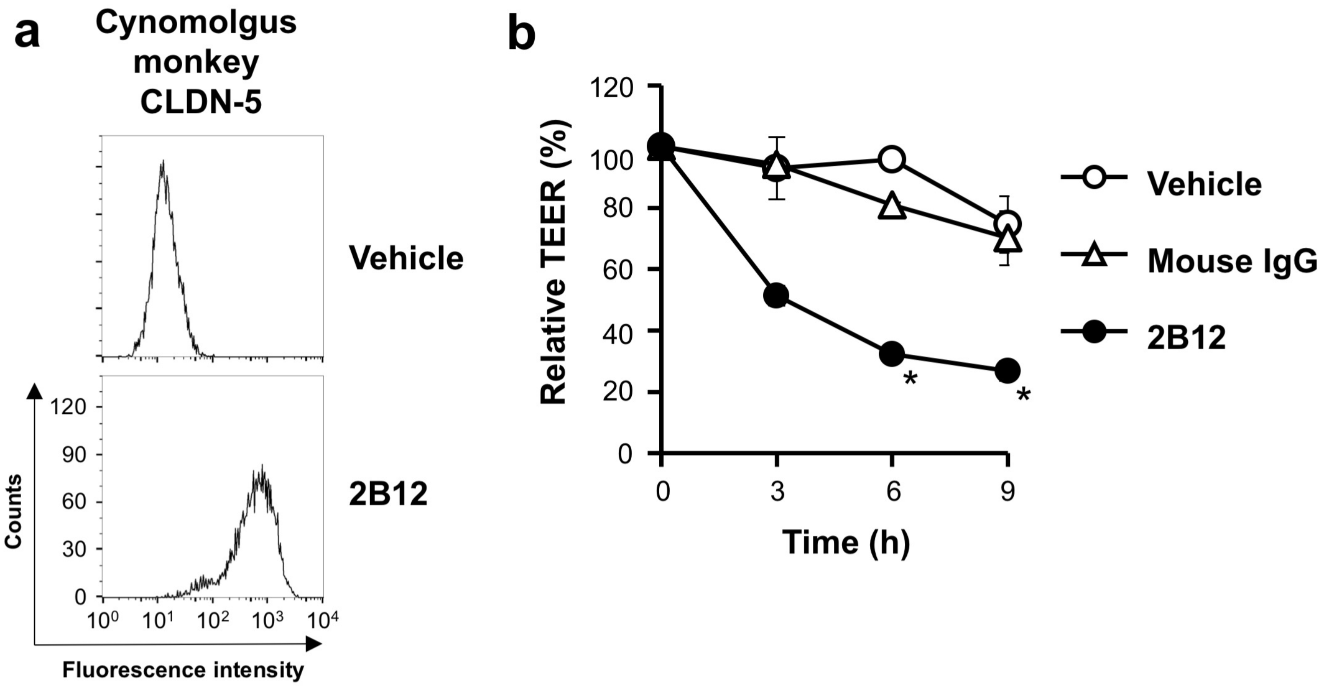

Supplementary Figure S3. Effect of anti-CLDN-5 ECR mAb clone 2B12 on monkey blood–brain barrier in vitro model. (**a**) Binding activity of anti-CLDN-5 ECR mAb clone 2B12 against cynomolgus monkey CLDN-5 expressing cells. Cynomolgus monkey CLDN-5–expressing HT-1080 cells were treated with vehicle (PBS) or 5 *μ*g/mL of 2B12. The cells were then treated with fluorescein-labeled secondary antibodies, and the fluorescently labeled cells were detected by means of flow cytometry. (**b**) TEER assay. Monolayers of monkey brain microvasculature endothelial cells were apically treated with vehicle (PBS) or 500 *μ*g/mL of antibodies (mouse IgG or 2B12) for 9 h. TEER was monitored throughout the treatment period. Data are expressed as percent TEER relative to the value at 0 h. Data are presented as mean ± SD (n = 3). *, P<0.05 versus vehicle treatment.
